# Impact of spatial characteristics in the left stenotic coronary artery on the hemodynamics and visualization of 3D replica models

**DOI:** 10.1038/s41598-017-15620-1

**Published:** 2017-11-13

**Authors:** Yang Yang, Xin Liu, Yufa Xia, Xin Liu, Wanqing Wu, Huahua Xiong, Heye Zhang, Lin Xu, Kelvin K. L. Wong, Hanbin Ouyang, Wenhua Huang

**Affiliations:** 1 School of Basic Medical Science, Southern Medical University, Guangzhou, Guangdong, China; 2 Guangdong Engineering Research Center for Translation of Medical 3D Printing Application, Guangzhou, Guangdong, China; 3 Guangdong Provincial Key Laboratory of Medical Biomechanics, Guangzhou, Guangdong, China; 4 Shenzhen Institutes of Advanced Technology, Chinese Academy of Sciences, Shenzhen, Guangdong, China; 5 Department of Ultrasound, The Second People’s Hospital of Shenzhen, Shenzhen University 1st Affiliated Hospital, Shenzhen, 518029 China; 6 Department of CardiologyGuangzhou, General Hospital of Guangzhou Military Region, PLA, Guangzhou, China; 7 School of Medicine, Western Sydney University, Sydney, Australia

## Abstract

Cardiovascular disease has been the major cause of death worldwide. Although the initiation and progression mechanism of the atherosclerosis are similar, the stenotic characteristics and the corresponding medical decisions are different between individuals. In the present study, we performed anatomic and hemodynamic analysis on 8 left coronary arterial trees with 10 identified stenoses. A novel boundary condition method had been implemented for fast computational fluid dynamics simulations and patient-specific three-dimensional printed models had been built for visualizations. Our results suggested that the multiple spatial characteristics (curvature of the culprit vessel multiplied by an angle of the culprit’s vessel to the upstream parent branch) could be an index of hemodynamics significance (r = −0.673, P-value = 0.033). and reduction of the maximum velocity from stenosis to downstream was found correlated to the FFRCT (r = 0.480, p = 0.160). In addition, 3D printed models could provide accurate replicas of the patient-specific left coronary arterial trees compare to virtual 3D models (r = 0.987, P-value < 0.001). Therefore, the visualization of the 3D printed models could help understand the spatial distribution of the stenoses and the hand-held experience could potentially benefit the educating and preparing of medical strategies.

## Introduction

Cardiovascular disease (CVD) is the leading cause of death globally regardless of sex that responding to approximately 30% of the mortality according to the Centers for Disease Control and Prevention (CDC) and World Health Organization^1,2^. The major factor contributes to the morbidity of CVD is atherosclerosis, which results in the stenosis in the artery that lead to ischemic symptoms, including myocardial infarction, and even stroke^3^. The severity of the stenosis is an important index for disease management. Despite improvements in precise diagnosis and intervention techniques, incidence of death due to coronary artery disease continues to increase by 40% for the last decade in China^4^. For symptomatic patients, the therapeutic procedures are effective in quality of life preservation and survival rate, but it is not the case for non-symptomatic patients until the occurrence of the acute symptoms that increase the risk of morbidity and mortality. Therefore, the comprehansive examinations are critical for screening the CVD risk in high-risk population to improve the survival rate and treatment management.

The computed tomography coronary angiography (CTCA) had emerged as one effective method for the diagnosis of the presence of clinical relevant coronary artery disease in low dose radiation^5^. Previous studies have proved the diagnostic accuracy of computed tomography angiography (CTA) for identifying severity of the stenosis and the capability for predicting acute coronary symptoms^6–8^, but the frequent overestimation of the hemodynamic significance causes excessive burden to the patient without obstructive CVD including economic and excessive treatment^9–11^. In addition to the diagnosis of the stenosis severity based on the morphologic identifications, the fraction flow reserve (FFR) had arisen to achieve the more accurate functional assessment of the vascular bed^12^. Although the FFR guided percutaneous coronary intervention (PCI) had benefited survival rate of the stable CAD^13,14^, the application rate of FFR is limited due to the invasive nature and the expense of the procedure^15^ that it was less than 10% of the CAD population in developed countries^16^. Consequently, CTA based fractional flow reserve (FFRCT) allow noninvasive assessment, providing 86% accuracy of diagnosing the ischemia-related stenosis and predicting the susceptible locations to the occurrence of atherosclerosis^17^. The hemodynamics analysis illustrated flow distribution variations due to the complex plaques for the assessment of the severity, but the visualization of the pathological arterial segments is limited in computer screens^18,19^ that make it difficult to assist in the preparation of the surgery planning without further invasive examination, such as the preparation of the stent before having Invasive coronary angiography (ICA).

Three-dimensional printing has been applied in cardiovascular to visualize anatomical structures for surgeons^20–22^. Improvement of the segmentation techniques facilitates the virtual visualization, but hands-on experience could deliver a direct impression on the anatomy. The gap between image and real-life experience had been bridged with the emerge of 3D printing. As the patient-specific anatomy of the vascular varied among individuals, tools and routes for revascularization would differ accordingly to have a better prognosis. The application of 3D printing in preoperative planning had optimized the endovascular repair of abdominal aortic aneurysm and aortic dissection in vascular surgery by assessing the risk of calcified plaque and developing the custom-made device for foreseen complications^23–25^.

In the present study, we implemented both hemodynamics analysis and 3D printing to patient-specific stenotic vessels for a comprehensive understanding of the relationship between spatial characteristics and hemodynamics variations. Since the limited correlation between severity of isolated stenosis and ischemia was found in previous studies, the combination effects of multiple spatial characteristics on the hemodynamics were evaluated. Validation of the calculation was performed by referring the calculated FFRCT to the invasive FFR measurements. Three-dimensional printing was applied in developing the hand-held model, in addition to the hemodynamics analysis by computational fluid dynamics(CFD) to provide a visual identification of the correlation between spatial characteristics and the hemodynamics in the culprit’s vessels.

## Method

### Data acquisitions

Patients were retrospectively identified who had undergone CTA and FFR from March 5, 2013, to April 22, 2015. The ethical review committee of Shenzhen Sun Yat-Sen cardiovascular hospital (Shenzhen, Guangdong, China) approved this research with waived informed consent and anonymized data was used for analysis. The exclusion criteria were as follows: occlusion in the culprit’s vessel; previous myocardial infarction; ACS in the previous 60 days; injection of adenosine or iodine-based contrast media were unavailable; had arrhythmia; undergone coronary artery bypass graft surgery or percutaneous coronary intervention previously. Therefore, there were 8 patients who were included in the present study. The age ranged from 59 to 74 years old (average 67 years old ± 17 years). CTA performed on the symptomatic patient who suffered from stable coronary artery disease following the Society of Cardiovascular Computed Tomography guidelines^26^. The CTA data during the diastolic phase were recorded for the anatomic analysis followed by the invasive FFR for functional assessment. Hyperemia was induced by using an infusion of adenosine (140 μg.kg-1.min-1) via the femoral vein. Then, a pressure-temperature sensor guidewire was placed through the catheter at the distal of the stenosis for the measurement of the arterial pressure. The FFR value was calculated as the ratio of the mean distal coronary pressure (mPd) to the mean aortic pressure (mPa) during hyperemia (Eq. 1).

1 $${\rm{FFR}}=\frac{{\rm{mPd}}}{{\rm{mPa}}}$$

### Model establishment

Eight subjects were selected for analysis and 3-dimensional printing. A previous study suggested that a simplified coronary arterial model without the component of the aorta could provide reliable hemodynamics analysis efficiently^27^. Therefore, the simplified geometric models of the coronary arteries were reconstructed from the patient-specific CTA image data and 10 culprit vessels were identified by the anatomic analysis. Nine out of the ten stenoses were considered above 50%. The details of the patient-specific arterial geometries were determined by the images and the arterial lumens were reconstructed for the CFD analysis and 3D printing with commercial software Mimics (Materialise NV, Leuven, Belgium). The nonstructural tetrahedron elements were used to generate meshes and the mesh-independent tests were performed. The densities of meshes were ranged from coarse (approximately 17,100 nodes with 85,600 elements) to fine (approximately 32,800 nodes with 545,820 elements) and the convergence was achieved when the calculated maximum velocities at the stenosis between two densities of meshed were lower than 0.1%. Since the volumes of the geometries varied individually, the average element numbers of all cases were 598,371 ± 103,490.

### Configuration of the computational fluid dynamics analysis

Boundary condition of the simulation played an important role in reproducing physiological hemodynamics. Lumped parameter model had been used to depict a reliable boundary condition, which consist of resistances and capacitance responding to the resistance to the blood flow and the compliance of the vascular bed^28^. A novel boundary condition method was applied for more effective calculations^29^. In brief, the mean flow rate to the coronary arteries was calculated based on myocardial mass^30,31^. The total resistance was then calculated as the ratio of mean aortic pressure and mean flow rate. The resistance of each outlet was derived from the total resistance of the arterial tree based on the scaling law^32^. Then the resistance to each outlet was calculated according to the mass conservation law that the outflow of all outlets equaled to the total flow rate. Since the resistances at the microvasculature were found to be independent of the stenosis severity^33^, the resistances of the outlets in stenotic arteries were the same to the normal arteries. Non-slip and rigid arterial wall was assumed. Assumptions of the blood flow were made as follows: incompressible, laminar and Newtonian^34^, the viscosity and density were constant at 0.0035 Pa.s and 1056 kg/m^3^, respectively. The flow momentum and mass conservation were solved by the Navier-Stokes governing equations:

2 $${\rm{\rho }}(\frac{{\rm{du}}}{{\rm{dt}}}+{\rm{u}}\cdot \nabla {\rm{u}})=-\nabla {\rm{p}}+{\rm{\mu }}{\nabla }^{2}{\rm{u}}+{\rm{f}}\,$$

3 $$-\nabla \cdot {\rm{u}}=0$$

where ρ is the density of blood, u is the velocity field, p is the pressure, μ is the viscosity, f was taken to be zero. Simulations were performed using COMSOL Multiphysics^35^.

### 3D printing of the coronary arteries

For the fabrication of the patient-specific models of coronary artery segments, the stereolithography (SLA) technique was used which is considered the gold standard in 3D model production with high resolutions of up to 0.025 mm^36^. The resolution of the printer (Prismlab RP 400, GDi, Shanghai, China) is 0.075 mm with smooth surface instead of “step” edge that was capable of forming the precise replica of patient-specific coronary arterial models with the scale of 1 mm to 4 mm of the cross-sectional area. The models were printed with liquid resin and the excess raw materials and the supporting structures were manually removed to accomplished the formation of the 3D printing models. Since the scale was small, several coronary artery segments were printed spontaneously that it took less than 2 hours to complete printing all models. This method allows the illustration of the complex positive remodeling of the stenosis that causes reduction of the lumens along the vessel. We fabricated our 3D printing models in collaboration with the Institute of anatomy, Southern Medical University, Guangzhou, China.

### Spatial characteristics and the hemodynamics

Spatial characteristics were quantified from the image-based virtual 3D models. These characteristics include severity of stenosis, locations of the lesions, the angle of the bifurcation at immediate upstream and the angle of the culprit’s vessel, the curvature of the bifurcations. The severity of the stenosis is calculated by the ratio of the diameter of the remaining lumen at the stenosis (DStenosis) and the healthy proximal segment (DProximal), (S = DStenosis/DProximal). Curvature (Cu, unit: 1 /m) of the culprit’s vessel was calculated from the segment between the coronary ostium and the immediate downstream of the stenosis. Locations of the lesions were determined by the parameters including distance from the coronary ostium and the eccentricity. The angle of bifurcations (Angle B) and the angle of culprit’s vessel (Angle C) were measured from the virtual 3D models. The immediate downstream of the lesion was measured at the immediate distal edge of the lesion (unit: degree). Mean pressure along each branch of the arteries were extracted from the calculations and the FFRCT along the vessel were calculated following Eq. 1. Accuracy of the FFRCT was validated by comparing to the invasive FFR measurement. Wall shear stress (WSS) was considered an important risk factor for the initiation and the progression of the plaque^37^, Therefore, the correlation of the spatial characteristics to the WSS was also investigated.

### Statistical analysis

**Figure 1:**
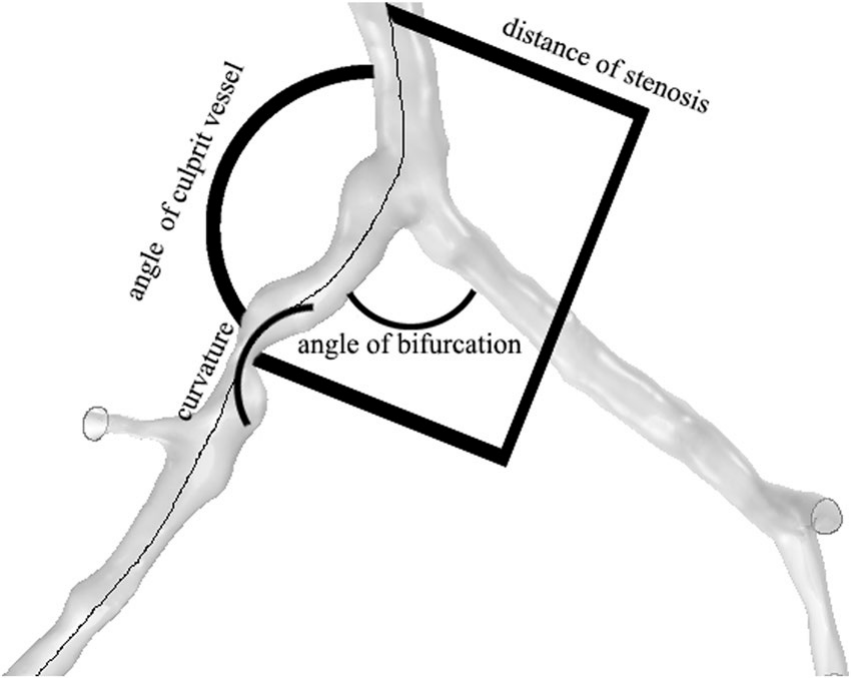
The schematics of the measurement of the spatial characteristics.

Spearman’s correlation was performed to investigate relationships between geometric characteristics to the severity of the stenosis, velocity and pressure. These characteristics included severity, angles of the bifurcation and the artery, distance of the stenosis from the ostium (Fig. 1). Bland-Altman plots were used to compare FFR measured by thermodilution with CFD-derived FFRCT to validate the accuracy of the calculations. Statistical analyses were performed using and SPSS (version 15, SPSS, Chicago, IL). Two-sided P values of <0.05 were considered significant.

## Results

**Figure 2:**
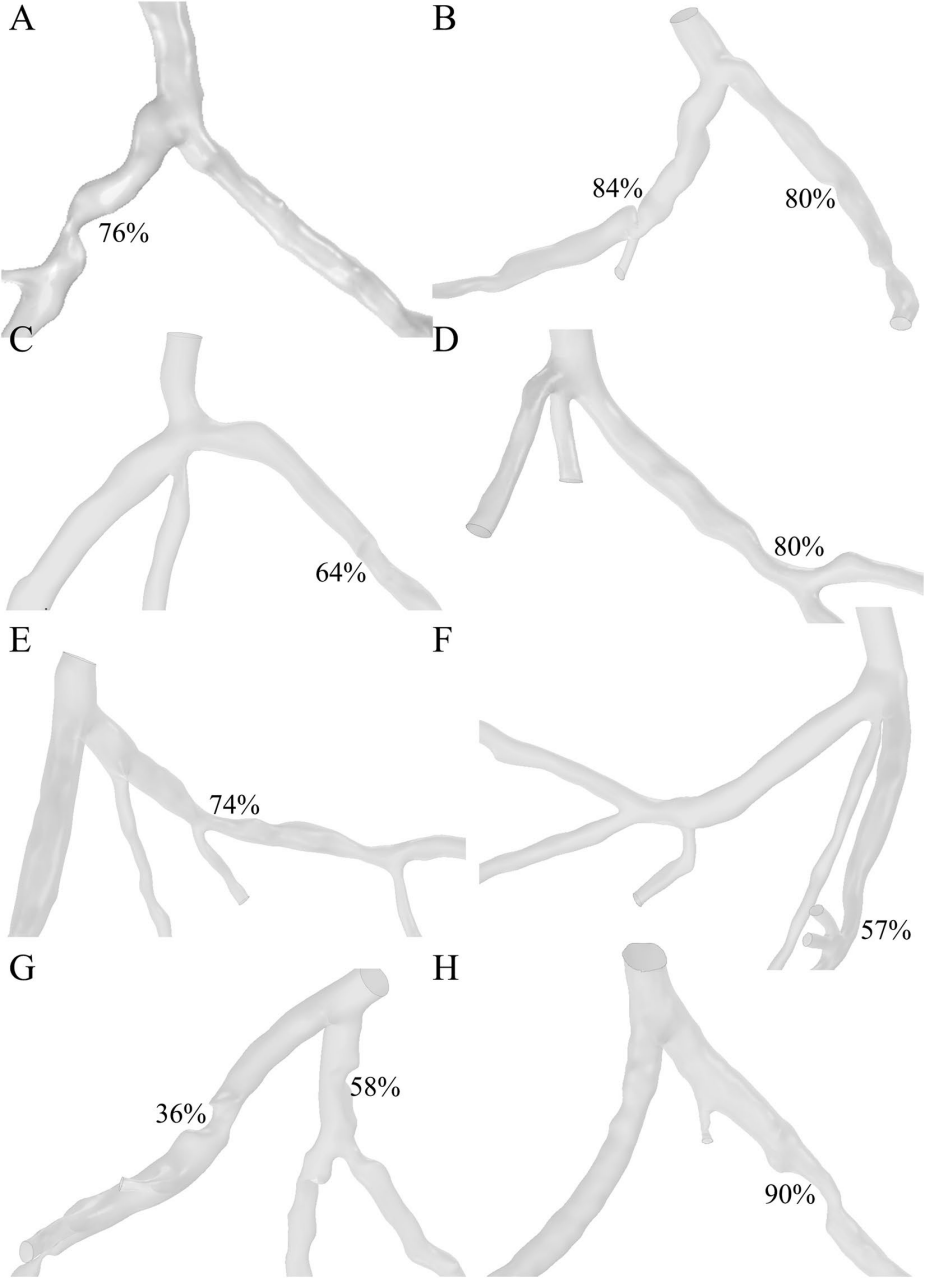
Eight patient-specific left coronary arterial trees were reconstructed from the computed tomography angiography. Ten stenoses were identified through the clinical practice. Only the lumens of the coronary arteries were reconstructed. The spatial characteristics, including severity of stenosis, locations of the lesions, the angle of the bifurcation at immediate upstream and the angle of the culprit’s vessel, curvature of the bifurcations, were measured from the virtual 3D models.

**Figure 3:**
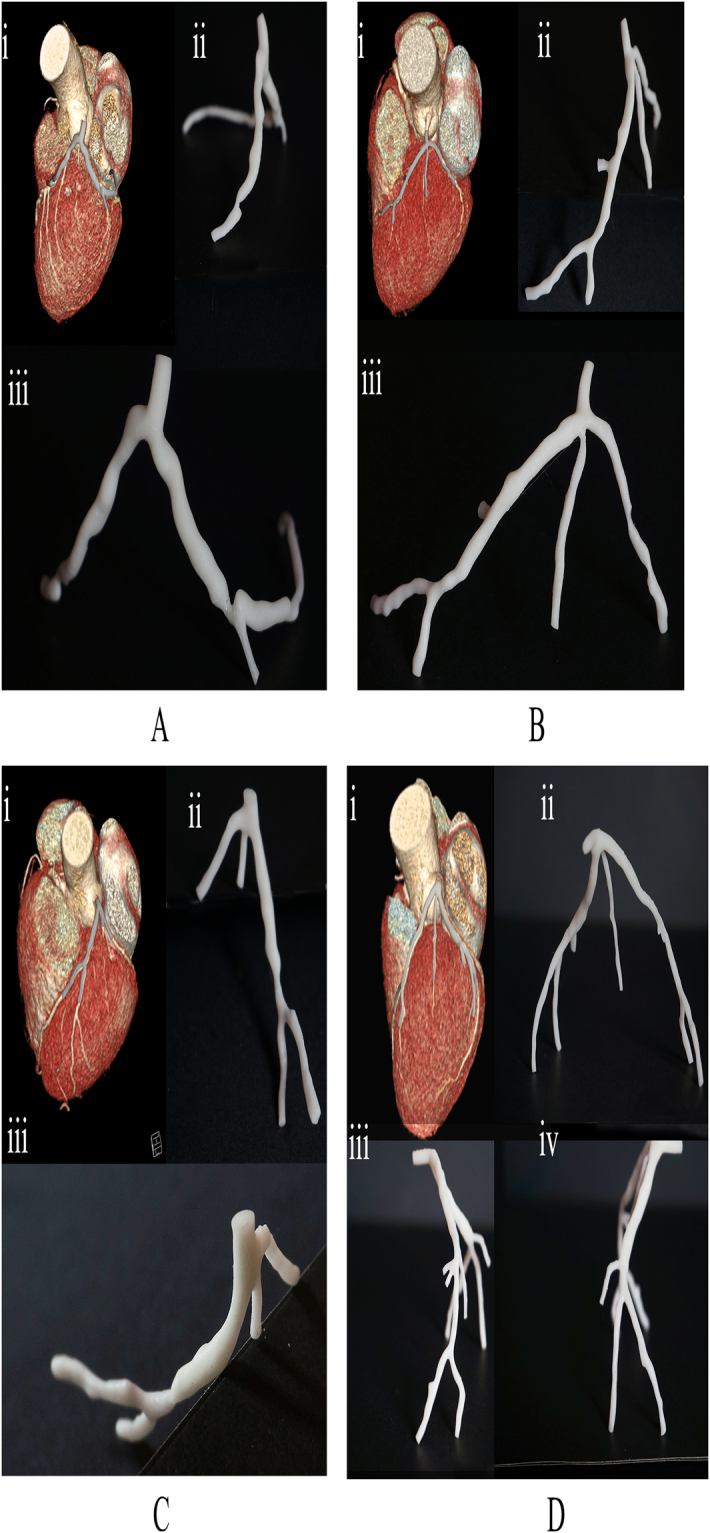
Three-dimensional printed models of the patient-specific left coronary arterial trees. i: The 3D printed models were overlapped on the virtual 3D models for comparison of the spatial characteristics. ii, iii, iv : The hand-held experience of the 3D printed models from different viewing angle.

**Figure 4:**
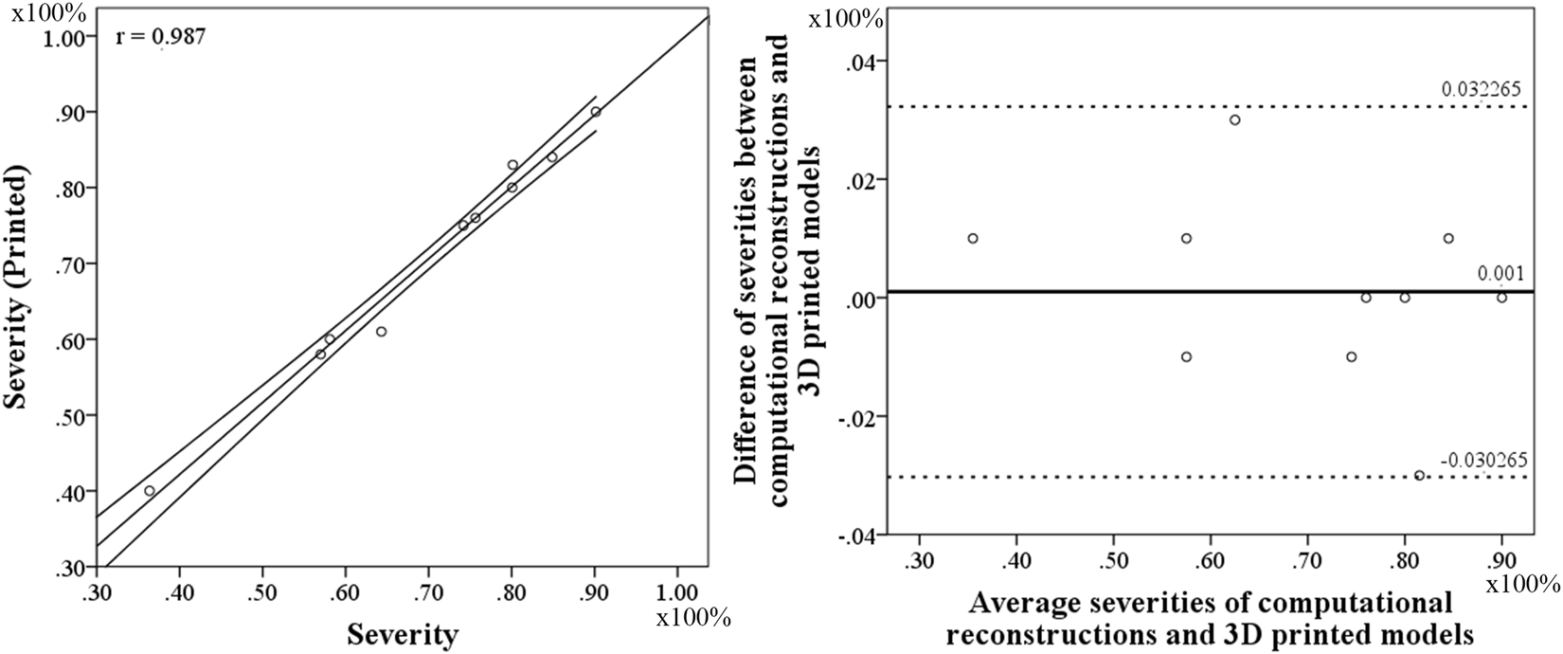
Evaluation of the accuracy of the 3D printed models. The anatomic severity of the stenosis was taken as an index. Correlation test showed good agreement between the severities of virtual 3D models and 3D printed models (left, r = 0.987 with 95% confident interval). Bland-Altman test showed consistency of the severities between virtual 3D models and 3D printed models (right, mean error = 0.1%).

We had reconstructed 8 patient-specific coronary artery geometries based on the patient-specific CTA images (Fig. 2). Ten stenoses from the 8 left coronary arteries were examined in the clinical practices. Measurement of the spatial characteristics was taken from the image-base virtual 3D models (Detail values are illustrated in Table 1). The Spearman test showed that curvatures of the arterial branches were negatively correlated with the severity of the stenosis (r = −0.707, P-value = 0.011). The angle of the upstream bifurcation and the angle of the culprit’s vessel to the parent vessel was independent of the severity of the stenosis (r = 0.085, P-value = 0.407 and r = −0.127, P-value = 0.363, respectively). The distance of the stenosis from the ostium of the coronary artery was significantly positively correlated with the severity of stenosis (r = 0.624, P-value = 0.027). For hand-held visualization, the three-dimensional patient-specific coronary arterial geometries had been printed (Fig. 3). The comparison between 3D printed models and the virtual 3D models was performed to evaluate the accuracy of the spatial characteristics of the 3D printed models. The stenotic severities were taken as the validate index. The diameters of the stenosis (DS) and the proximal segment (DP) were measured in each 3D printed model and the severity of the stenosis was the ratio of the DS to the DP. The correlation test showed that the 3D printed models were in good agreement with the virtual 3D models (r = 0.987, with 95% confident interval, Fig. 4) and the Bland-Altman test showed good agreement with a mean difference of 0.1% (Fig. 4).

**Table 1:** Detail values of the spatial characteristics of the patient-specific left coronary arterial tree virtual 3D models. Severity: severity of the stenosis; Distance: the distance from the ostium of the coronary arterial tree to the throat of the stenosis along the center line of the vessel (Unit: mm). Angle B: angle of the bifurcation of the upstream of the culprit vessel (Unit: degree). Angle C: angle between the culprit vessel and the upstream parent branch (Unit: degree). Curvature: the curvature of the culprit vessel (Unit: 1/m). Correlation to severity: the correlation factors between severity and the other spatial characteristics (with P-value below). Correlation to FFRCT: the correlation factors between FFRCT and the spatial characteristics (with P-value below).

	Severity	Distance [mm]	Angle B [degree]	Angle C [degree]	Curvature [1 /m]
	76%	25.79	90.73	39.04	0.3
	85%	52.71	92.05	43.51	0.23
	80%	42.69	92.05	48.54	0.23
	64%	27.75	120.16	78.11	0.21
	80%	28.49	92.88	20.84	0.13
	74%	32	93.36	61.64	0.16
	57%	37.5	80.37	36.42	0.65
	36%	14.21	62.17	27.98	0.37
	58%	18.7	62.17	45.93	0.37
	90%	34.09	58.81	20.89	0.1
Correlation to Severity	—	0.624	0.085	−0.127	−0.707
P-value	—	0.027	0.407	0.363	0.011
Correlation to FFRCT	−0.164	−0.03	0.128	0.067	0.079
P-value	0.326	0.467	0.362	0.427	0.414

**Figure 5:**
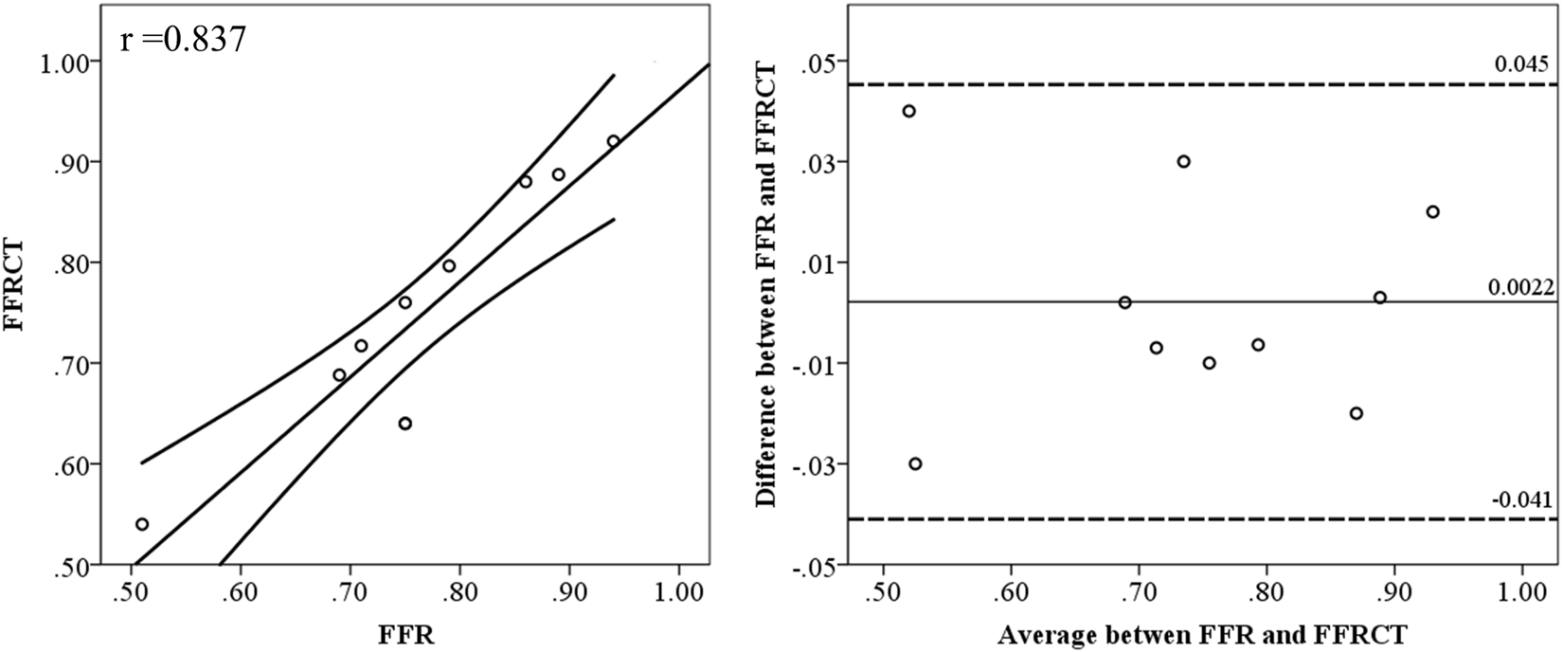
Evaluation of the accuracy of the FFRCT. The invasive FFR value was taken as an index. Correlation test showed good agreement between the invasive FFR and FFRCT (left, r = 0.837 with 95% confident interval) with the spearman correlation factor (r = 0.985, p < 0.01). Bland-Altman test showed consistency between invasive FFR and FFRCT (right, mean error = 0.0022).

Hemodynamics in the coronary arteries were depended on the spatial characteristics. Validation of the calculations was by comparing the calculated FFR to the invasive FFR (R = 0.837 with 95% confident interval as showed in Fig. 5) that good agreement had been achieved according to the Spearmen correlation test (R = 0.985, P < 0.01). The diversions of the calculations are lower than 0.0022 compared to the measurement according to the Bland-Altman test (Fig. 5).

**Figure 6:**
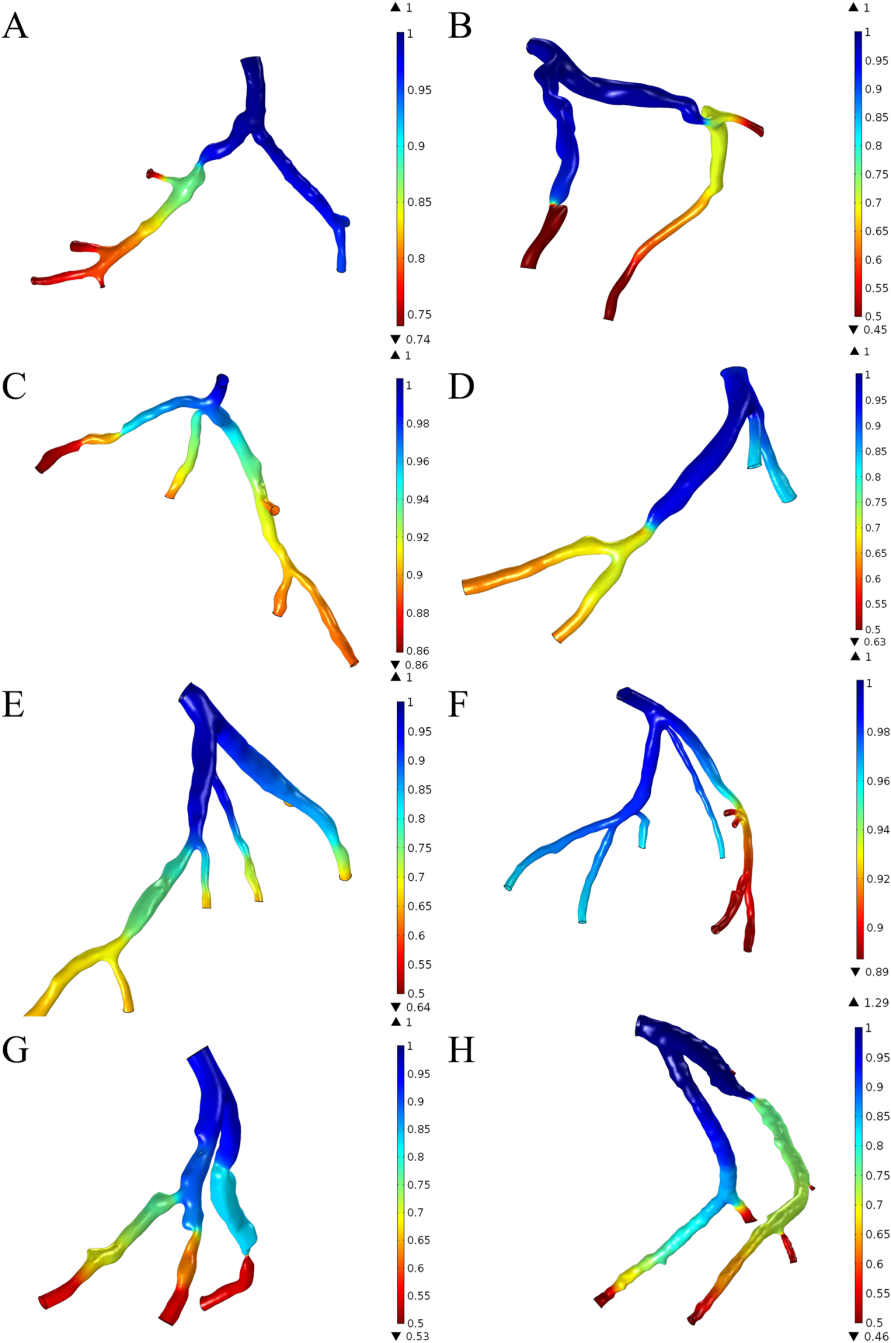
Pressure drop in the left coronary arterial trees. FFRCT values were found significantly decrease in the severe stenosis (**B**, **G**). Insignificant changes of FFRCT values in the bifurcation of the main branches and the mild to moderate stenoses (**A**, **C**, **D**, **E**, **F**, **H**). However, the weak correlation between FFRCT values and the severities of the stenosis was found (r = −0.164, P-value = 0.326).

The pressure drops at the severe stenosis were found responsible for the significant pressure drop (Fig. 6B,G). But the correlation to the severities was insignificant (r = −0.164, P-value = 0.326). Meanwhile, the spatial characteristics were found contributing insignificantly to the variation of the pressure distribution that correlation factors were low in general (P-value of the distance, angles, and curvature was 0.467, 0.362, 0.427, 0.414, respectively).

### Correlation between spatial characteristics and the hemodynamics

**Figure 7:**
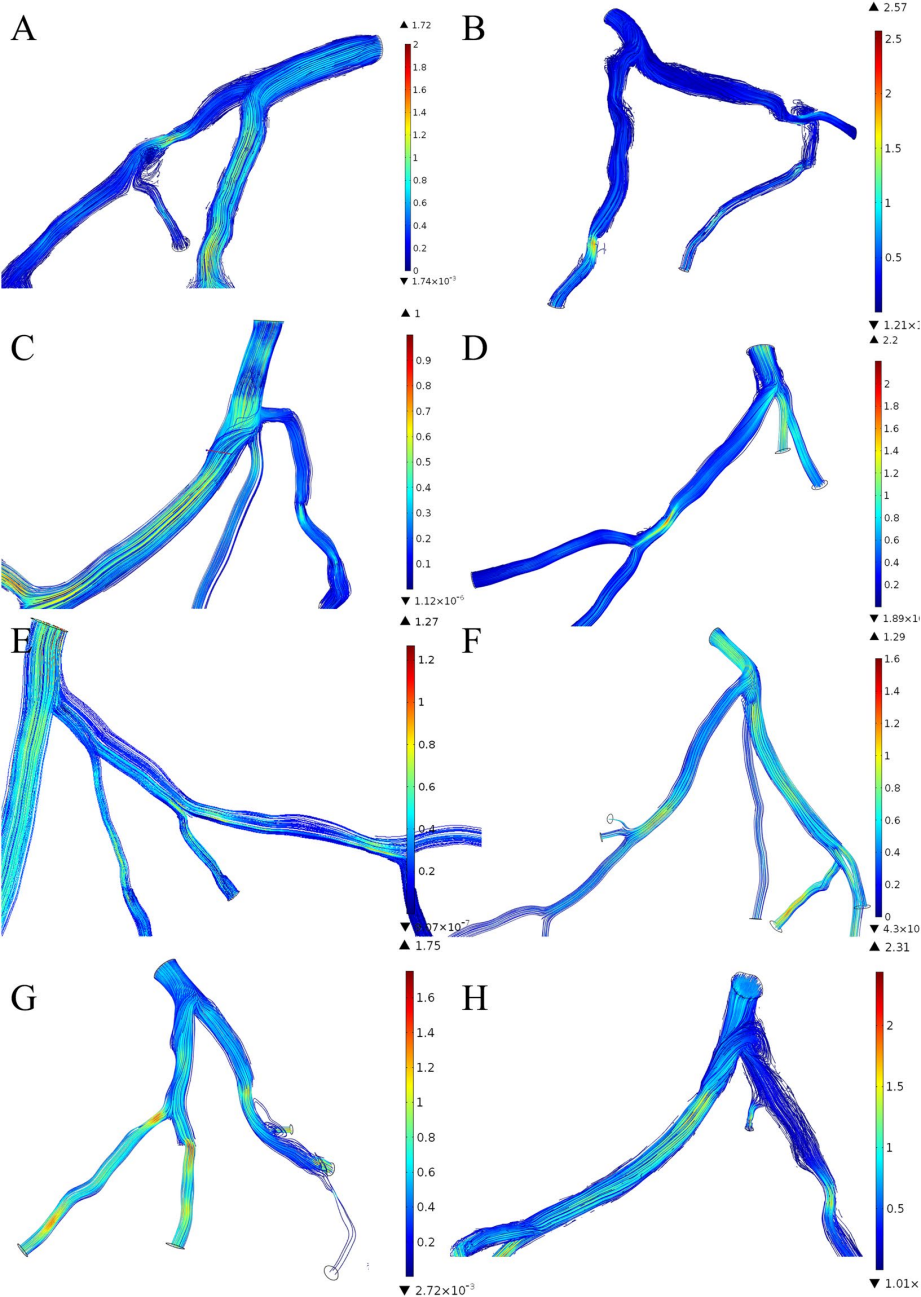
Velocity distribution in the left coronary arterial trees. The velocity accelerated at the stenoses. However, stenosis in the culprit’s vessel increased the resistance to the blood flow and the shunted the blood flow to the other child branch in consequence that caused disturbance of the blood flow in the upstream bifurcation of the culprit vessel and the other child branch (**A**, **C**, **D**, **E**, **G**, **H**) (Unit: m/s).

The stenosis in the coronary arteries was found correlated to the acceleration of the maximum blood flow velocity at the stenosis (Fig. 7). The velocity magnitudes increased compared to the upstream and downstream of the stenosis in the culprit’s vessel, the maximum velocities were usually found at the throat of the stenosis. However, severity of the stenosis was not correlated to the appearance of the maximum velocity (r = 0.486, P-value = 0.154) as well as the separated spatial characteristics (angle of the bifurcation: r = 0.545, P-value = 0.103; angle of the culprit vessel: r = −0.114, P-value 0.754; curvature: r = −0.422, P-value = 0.224). Therefore, the combined effect of the spatial characteristics was investigated. By multiplying the value of the spatial characteristics, the correlation test to the velocities was evaluated. The maximum velocities at the stenosis were found to be strongly correlated to the curvatures multiplied by angles of the culprit’s vessels (r = −0.673, P-value = 0.033). Weak correlations were found between maximum velocity to the curvature multiplied by severity (r = −0.419, P-value = 0.228) and angle of the culprit vessel multiplied by severity (r = −0.511, P-value = 0.132). The maximum velocity at the stenosis was found weak correlated to the FFRCT (r = 0.16, p = 0.75). However, the reduction of the maximum velocity from that of stenosis to the downstream was found correlate to the FFRCT(r = 0.480, p = 0.160).

### Recirculation and WSS distribution

Recirculation zones were found at the downstream of the stenosis (Fig. 7). Lengths of the recirculation zones in the coronary arteries were measured. The recirculation zones were found in 6 out of 8 coronary arteries except for Fig. 7E and F, of which the severity was 74% and 57%. The location of the stenosis was found responsible for the length of the recirculation zone. For Fig. 7A,D, the stenosis located at the parent branch proximal to the bifurcation and the corresponding length of the recirculation zone was 3.92 mm and 1.11 mm, respectively. For Fig. 7B,C,G,H, the stenosis located at the child branches downstream to the bifurcations and the corresponding length of the recirculation zone were 1.56 mm, 21.72 mm, 6.09 mm, 9.1 mm, and 10.7 mm, respectively. On the other hand, the blood flow was disturbed at the upstream bifurcation when stenosis was found at the child branch that the blood flow was diverted to the normal branch (as showed in Figs 7D and H).

**Figure 8:**
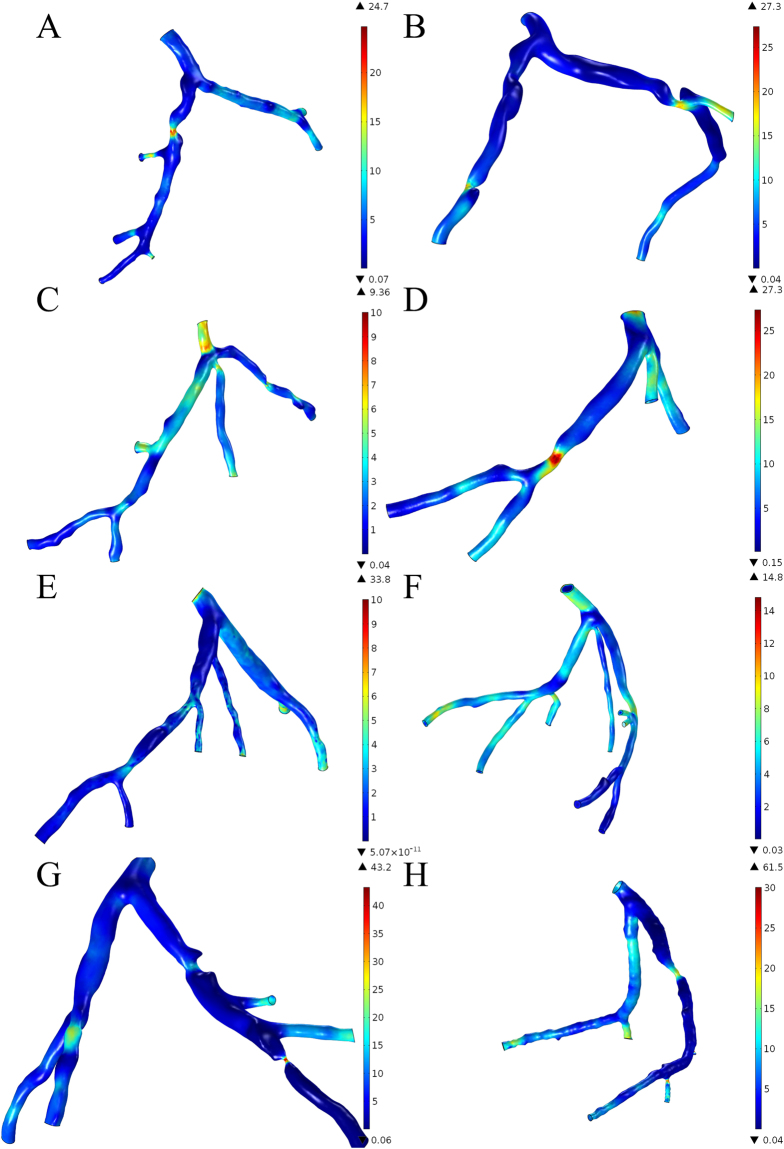
Wall shear stress (WSS) distribution in the left coronary arterial trees. High WSS were found at the throat of the stenosis in the culprit’s vessels. The mean standard deviation of the WSS at the stenosis was 19.52 ± 10.04 Pa (range from 5.2 Pa to 30.3 Pa). High WSS was also found in the normal branch distal to the bifurcation when the other child branch was culprit vessel (**A**, **C**, **D**, **E**, **F** and **H**).

Wall shear stress (WSS) distribution varied along the vessels (Fig. 8). High WSS were found at the throat of the stenosis in the culprit’s vessels. The mean standard deviation of the WSS at the stenosis was 19.52 ± 10.04 Pa (range from 5.2 Pa to 30.3 Pa). High WSS was also found in the normal branch distal to the bifurcation when the other child branch was culprit vessel (Fig. 8A,C–F,H).

## Discussion

This study was undertaken to provide a further understanding of the left coronary arterial geometries in a relationship with the hemodynamics. The spatial characteristics varied among individuals that caused patient-specific hemodynamics in the coronary arteries^38,39^. The current study investigated the contribution of the different spatial characteristics, such as curvature, the angle of the upstream bifurcation, the angle of the culprit’s vessel and the severity of the stenosis, to the distribution of pressure drop, WSS and flow velocity. We performed the calculation of the 3D patient-specific left coronary arterial geometries with identified stenosis and we performed 3D printing of the coronary arterial models for visualization of the spatial relationship of the coronary arteries. We found a correlation between maximum velocity at the stenosis and the combined contribution of curvature with the angle of the culprit’s vessel. In the present study, the main branches and the significant side branches of the left coronary arteries were included in the 3D virtual models. The major blood flow distribution were accounted for the hemodynamic distribution analysis. The isolated spatial characteristics were weak correlated to the hemodynamics variation, while negative correlation was found in distance and severity and positive correlation in bifurcation angles and curvature of the culprit vessel.The coronary arterial geometries are different individually, the development of the stenosis is, therefore, a consequence of the joint effect of multiple factors. We focus on the effect of spatial characteristics and we investigated the effect of the combination of different spatial characteristics. Our results imply that the combination of the curvature with the angle of the culprit’s vessel could be a potential index for the maximum velocity at the stenosis. Significant correlation was found between the combination of curvature with the angle of the culprit’s vessel to the maximum velocity at the stenosis (r = −0.673, P-value = 0.033). Increasing velocity is responsible to the increasing WSS on the plaque that the increasing biomechanical drag force is prompt to increase the risk of plaque rupture^40,41^, and the high WSS also induce specific changes in endothelial cell behavior, which leads to the progression of the atherosclerotic lipid core^42^. Moreover, the stenosis reduces the blood flow to the culprit’s vessel. The velocity in the non-culprit vessel are significantly increased compare to the culprit’s vessels and the flow to non-culprit vessels was generally higher than the culprit vessels (Fig. 7A,C,E–H). For Fig. 7B, stenoses were found on both child branches, the blood flow velocities are similar, yet, the flow to the less severe stenotic branch (left) was slightly higher than the other (left 80%: 3.47 ml/s, right 84%: 3.37 ml/s). For Fig. 7D, although the severity of the stenosis in the culprit’s vessel was 80%, the larger lumen diameter of the proximal culprit branch (8.58mm^2^) allowed more blood flow to the culprit vessel (2.04 ml/s) instead of the non-culprit vessel (1.01 ml/s). The additional flow to the non-culprit vessel also caused disturbance to the flow distribution. The WSS distribution indicated the flow disturbance of the flow on the vessel wall (Fig. 8). High WSS (>10 Pa) were distributed at the non-culprit child branch proximal to the bifurcation (Fig. 8A,C,G,H). Low WSS were found in Fig. 8D,F at the outer wall of the non-culprit vessel proximal to the bifurcations, which implies the disturbance of the blood flow that potentially highlights the location that prompted to initiate atherosclerosis^43^.

### 3D printing in facilitating the understanding of the spatial characteristics

The 3D printed models facilitated the sensory understanding of the spatial characteristics of the patient-specific left coronary arterial trees. The virtual illustrations of 3D models had advanced the understanding of the anatomy from 2D to 3D. The manipulation of the virtual arterial trees on the computer could provide quantitative measurements and the understanding of the relationship to the surrounding tissues. However, the complex anatomies of the stenotic arteries were difficult to understand despite the use of virtual 3D models^44^. Previous studies have shown that 3D printed models could improve quality in teaching anatomy and preparing surgery^45–47^. The 3D printed models in the present study were reliable in visualizing the severities of the stenoses. By relating the 3D printed models to the position in the heart (Fig. 3), the stenoses were prompted to form on the outer wall of the vessel in the proximal segments (Fig. 3A–D), while the on the inner wall of the vessel in the distal segment (Fig. 3A). By holding in hand, 3D printed models are more easily manipulated compare to the virtual 3D models. We implemented SLA techniques for 3D printing in the present study because of the fast fabrication and readily prepared in our lab. The comment method of 3D printing applied in the biomedical and clinical evaluations were: i) selective laser sintering (SLS), (ii) stereolithography (SLA) [6], (iii) fused deposition modeling (FDM), and (iv) Poly jet modeling. While SLS and SLA are typically faster than FDM and PBE, they both require lasers and optics^36,48^. Comparing the structural integrity, and ability to fabricate complex structures, SLS and Poly jet modeling could produce high accuracy model with complex surface with an additional advantage to print in multiple materials. However, the cost-efficient was low compare to SLA and potentially hazardous in SLS would require extract cautions in application. The high cost-efficient in FDM is notable, but it is not suitable in printing complex shape due to the removal of the support could damage the delicate scale of coronary arterial models^36^.

### limitations

The limited sample size may have introduced uncertainties of the contributions from various spatial characteristics, but our results have implied the joint effects of multiple characteristics as a potential index to the ischemia-related stenosis. On the other hand, we noticed the differences between the overlapped 3D printed models and the image-based virtual 3D models (Figure 3Ai, 3Bi, 3 Ci, 3Di,). The diversions could be introduced by photographic angle or individual error during segmentations. The 3D printed models were illustrating the lumen geometries of the coronary arteries, which was directly related to the positive remodeling of the atherosclerotic plaque and lumen reductions. But the rigid material and the single color of the 3D printing may have limited the understanding of the state of plaque and hemodynamic distribution along the vessel. Further study may introduce flexible material and multiple color into the 3D printing to illustrate the WSS distribution and distensibility of the vessel for optimizing surgery strategy^49^.

## Conclusion

The different spatial characteristics among individuals lead to variation distribution of the stenoses, therefore specific understandings of the anatomic and hemodynamic distributions are required for patient-specific medical decision-making. In this study, we presented the analysis of the correlation of patient-specific left coronary arterial tree to the hemodynamics distributions. CFD simulations had been performed for hemodynamics analysis and 3D printed models had been built for visualization. Our results implied that multiple spatial characteristics combination could potentially improve the non-invasive evaluation of the culprit vessel. In additional, the 3D printed models could provide a hand-held experience that accurately produces the appearance of the coronary arterial trees in the *in vivo* condition and potentially reduces the need of cadavers for surgery training and practicing.

### Ethics approval and consent to participate

 The study was approved by the ethical review committee of Shenzhen Sun Yat-Sen cardiovascular hospital (Shenzhen, Guangdong, China) and was conducted in conformance with the Helsinki Declaration. Since this study is a retrospective study, the informed consent was waived and anonymized data was used for analysis.

### Availability of data and materials

The datasets analyzed during the current study are not publicly available due to the further analysis of the datasets being doing in our research but are available from the corresponding author on reasonable request.

### Declaration and verification

All the authors stated that the work described has not been published previously, that it is not under consideration for publication elsewhere, that its publication is approved by all authors and tacitly or explicitly by the responsible authorities where the work was carried out, and that, if accepted, it will not be published elsewhere in the same form, in English or in any other language, including electronically without the written consent of the copyright-holder.
